# Mechanistic insights into autocrine and paracrine roles of endothelial GABA signaling in the embryonic forebrain

**DOI:** 10.1038/s41598-019-52729-x

**Published:** 2019-11-07

**Authors:** Yong Kee Choi, Anju Vasudevan

**Affiliations:** 1 Department of Psychiatry, Harvard Medical School, Boston, MA 02215 USA; 2 Angiogenesis and Brain Development Laboratory, Division of Basic Neuroscience, McLean Hospital, 115 Mill Street, Belmont, MA 02478 USA

**Keywords:** Neuro-vascular interactions, Neuronal development, Developmental neurogenesis

## Abstract

The developing cerebral cortex uses a complex developmental plan involving angiogenesis, neurogenesis and neuronal migration. Our recent studies have highlighted the importance of endothelial cell secreted GABA signaling in the embryonic forebrain and established novel autonomous links between blood vessels and the origin of neuropsychiatric diseases. A GABA pathway operates in both endothelial cells and GABAergic neurons of the embryonic telencephalon; however, while the neuronal GABA pathway has been extensively studied, little is known about the endothelial GABA pathway. Our recently generated *Vgat* endothelial cell knockout mouse model that blocks GABA release from endothelial cells, serves as a new tool to study how endothelial GABA signaling shapes angiogenesis and neurovascular interactions during prenatal development. Quantitative gene expression profiling reveals that the endothelial GABA signaling pathway influences genes connected to specific processes like endothelial cell proliferation, differentiation, migration, tight junction formation, vascular sprouting and integrity. It also shows how components of the neuronal GABA pathway, for instance receptor mediated signaling, cell cycle related components and transcription factors are affected in the absence of endothelial GABA release. Taken together, our findings delineate the close relationship between vascular and nervous systems that begin early in embryogenesis establishing their future interactions and interdependence.

## Introduction

GABA is well established as the first excitatory transmitter to become functional in the embryonic brain and exerts diverse region-specific roles at different developmental stages. It is a deeply interesting and versatile molecule. Multi-modal actions of GABA-GABA_A_ receptor signaling have been elucidated in individual cortical layers during embryonic brain development^1^. It plays an important role in building the cortical network by controlling processes like neural progenitor proliferation, neuronal migration, dendritic maturation and synaptogenesis^2–4^. Abnormalities in neurons, altered GABA_A_ receptor distribution and dysregulated tonic inhibition have been implicated in neuropsychiatric diseases such as epilepsy, autism, schizophrenia and depression^1,5–11^. However, traditional views of neocortical development have depicted this source of GABA to be exclusively neuronal. We believe that GABA’s versatility is due to its different sources in the embryonic forebrain - neuronal versus endothelial with select roles performed depending on the cell type that secretes it. Our discovery of a novel GABA and its receptors’ signaling pathway within forebrain endothelial cells independent of the traditional GABA neuronal pathway has produced a new way to think about the origin and mechanisms of psychiatric illnesses^12^. The vesicular GABA transporter (*Vgat*) loads GABA from the endothelial cytoplasm into vesicles. By deleting *Vgat* specifically from endothelial cells, we were able to successfully turn off endothelial GABA secretion during embryonic brain development, illustrating that *Vgat* is the primary mechanism for GABA release from endothelial cells at early embryonic stages^12^. No GABA transporters (GATs) were present at this stage in telencephalic endothelial cells. Thus, we were able to evaluate the full significance of *Vgat* in endothelial GABA release for angiogenesis as well as other key cellular events during forebrain development - neurogenesis, radial migration of projection neurons and tangential migration of GABAergic interneurons, all of which were affected to some degree. With respect to angiogenesis, the cellular events that were significantly affected were endothelial cell proliferation, migration, tube formation and loss of tight junction formation with increased vascular permeability^12^. These results indicated that endothelial GABA signaling is connected to other key angiogenesis pathways. With respect to fostering neuro-vascular interactions, endothelial cell secreted GABA played a critical role for long distance GABAergic neuronal migration in the embryonic forebrain^12^. Neuronal GABA was unable to compensate for this unique role of endothelial GABA, since GABAergic neuronal tangential migration was significantly affected in the absence of endothelial GABA. The *Vgat* endothelial cell conditional knockout (*Vgat*^*ECKO*^) mouse model developed severe seizures during the early postnatal period and did not survive beyond 2 months of age^12^. Thus, this endothelial Vgat-GABA signaling pathway is a key mediator of vascular development and neuro-vascular interactions in the prenatal period, important for cortical circuit formation and indispensable for proper behavioral function.

As we now understand the significance of this vascular GABA pathway, it is essential to gain mechanistic insights by segregating this pathway specifically in endothelial cells and to see how neuronal gene expression is affected in the absence of endothelial GABA signaling. Global gene expression profiling of whole brain is not sufficient to address this issue and cell-type specific isolation of endothelial cells versus neuronal cells is important to achieve this goal. For instance, what molecular changes occur in telencephalic endothelial cells in the absence of endothelial *Vgat* and subsequent GABA release? How does loss of vascular GABA release affect genes that are critical for neuronal development? The *Vgat*^*ECKO*^ mouse model is ideal to address such questions. It is significant for identifying not only the mechanistic basis by which endothelial cell secreted GABA achieves its autocrine actions on angiogenesis and paracrine actions on neurons, but also, it serves as new resource, to isolate hitherto unknown or novel molecular mechanisms that regulate vascular development in the embryonic forebrain.

By using microarray technology, we were able to profile periventricular endothelial cells and neuronal cells from control (*Vgat*^*fl/fl*^) and *Vgat*^*ECKO*^ embryonic telencephalon at high resolution. Our results show that extensive molecular changes occurred within the telencephalic endothelium and within neuronal cells due to loss of endothelial GABA release. It provides valuable insights into altered expression of transcription factors, Wnt signaling and tight junction molecules as well as new GABA signaling components in *Vgat*^*ECKO*^ endothelial cells. Additionally, it shows how gene expression programs that regulate neuronal proliferation, differentiation and migration are altered. Furthermore, our study explains how changes in cell-type specific gene expression are a key determinant of behavioral outcome.

## Results

### Isolation of endothelial and neuronal populations from *Vgat*^*ECKO*^ telencephalon and gene expression profiling

**Figure 1:**
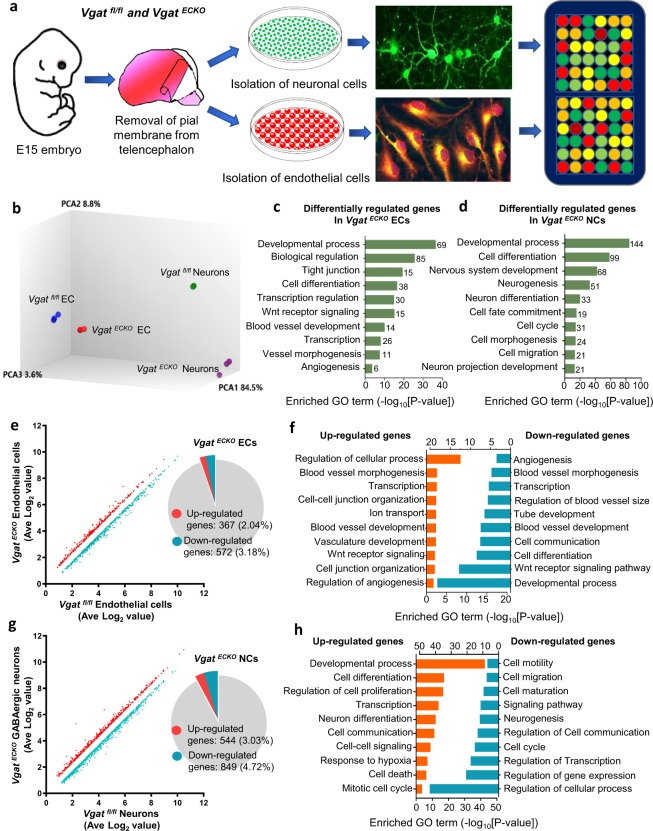
Comparison of gene expression profile of *Vgat*^*fl/fl*^ and *Vgat*^*ECKO*^ endothelial and neuronal cells. (**a**) Schematic depicting brain dissection, removal of pial membrane, isolation and culture of periventricular endothelial cells and neuronal cells from E15 *Vgat*^*fl/fl*^ and *Vgat*^*ECKO*^ mouse embryos and subsequent microarray hybridization. (**b**) PCA analysis of gene expression profile from endothelial cells and neuronal cells, n = 3 biological samples. (**c**,**d**) Gene ontology analysis of differentially expressed genes in *Vgat*^*ECKO*^ endothelial cells (**c**) and neuronal cells (**d**). (**e**) Scattered plot and pie chart (inset) expression for up- and down-regulated genes in E15 *Vgat*^*ECKO*^ endothelial cells compared to *Vgat*^*fl/fl*^ endothelial cells. (**f**) GO biological process analysis of up- and down-regulated genes in *Vgat*^*ECKO*^ endothelial cells. (**g**) Scattered plot along with pie chart expression for up- and down-regulated genes in *Vgat*^*ECKO*^ neuronal cells when compared to controls. (**h**) GO biological process analysis of up- and down-regulated genes in *Vgat*^*ECKO*^ neuronal cells. ECs: endothelial cells; NCs: neuronal cells.

To understand in detail how endothelial GABA modulates the neuronal GABA signaling pathway, we isolated and cultured periventricular endothelial cell and neuronal cell populations from *Vgat*^*fl/fl*^ and *Vgat*^*ECKO*^ telencephalon at embryonic stage 15 (E15), an age midway through mouse cortical neurogenesis and migration, using well established methods^13–16^. In the *Vgat*^*ECKO*^ model, endothelial GABA signaling is affected from early embryonic stages. Therefore, *Vgat*^*ECKO*^ neurons isolated at E15 have already undergone significant molecular changes in the absence of endothelial cell secreted GABA *in vivo*. Since key cellular events were affected in both cortical and sub-cortical telencephalon by this stage^12^, we did not restrict the neuronal isolation spatially. The neuronal population was characterized by immunocytochemistry, morphology, and sub-type specific marker expression and was found to be predominantly GABAergic, with a small proportion of glutamatergic neurons and an absence of microglia (Supplementary Figures 1 and 2). RNA was isolated from cell type specific samples and subsequent microarray hybridization and analysis was performed (Fig. 1a). Principal component analysis (PCA) showed differences in molecular signatures between endothelial cells and neurons from *Vgat*^*ECKO*^ and *Vgat*^*fl/fl*^ telencephalon based on cell segregation, that were further supported by strict clustering of independent triplicates (Fig. 1b). Endothelial or neuronal cells were also well distinguished by their genotypes (*Vgat*^*fl/fl*^ or *Vgat*^*ECKO*^) by PCA 2 and 3 in the 3D PCA table (Fig. 1b). Differential gene expression analysis provided a robust identification of changes in several distinct gene ontology (GO) terms/pathways in *Vgat*^*ECKO*^ endothelial and neuronal cells (Fig. 1c,d), including ‘tight junction’ and ‘Wnt receptor signaling’ categories in endothelial cells versus ‘neurogenesis’, ‘cell fate commitment’ and ‘cell migration’ categories in neuronal cells. Analysis of differentially expressed genes depicted global up-and downregulation in both endothelial cells and neuronal cells from *Vgat*^*ECKO*^ telencephalon (Fig. 1e,g). These variations in gene expression were graphed using a scatter plot and a pie chart to distinguish between up (orange) and down (blue) regulated genes in *Vgat*^*ECKO*^ endothelial and neuronal cells (Fig. 1e,g). We found 367 (2.04%) or 572 (3.18%) significantly up- or down-regulated genes in *Vgat*^*ECKO*^ endothelial cells (Fig. 1e) and 544 (3.03%) or 849 (4.72%) up- or down-regulated genes in *Vgat*^*ECKO*^ neuronal cells with comparison to controls (Fig. 1g). GO analysis with up- or down-regulated gene set of *Vgat*^*ECKO*^ periventricular endothelial cells revealed that terms such as ‘developmental process’, ‘Wnt receptor signaling’, ‘blood vessel development’, ‘blood vessel morphogenesis’, and ‘angiogenesis’ were strongly downregulated in *Vgat*^*ECKO*^ endothelial cells, in contrast to enrichment of GO term, ‘regulation of cellular process’, in the up-regulated gene set (Fig. 1f). This observation is consistent with the reduction in blood vessel densities and low angiogenic score observed in the *Vgat*^*ECKO*^ forebrain^12^. In neuronal sets, however, significant up-and down-regulation was observed in ‘developmental/cellular processes and ‘cell proliferation/cell cycle’ categories (Fig. 1h). This is also interesting since the *Vgat*^*ECKO*^ telencephalon showed an increase in mitotic cells in the SVZ while no differences were observed in the VZ, indicative of significant differential expression in cell cycle related genes^12^. *Vgat*^*ECKO*^ neurons also showed an increase in neuronal differentiation, hypoxia related and cell death categories (Fig. 1h) and a down-regulation in cell maturation, motility and migration categories (Fig. 1h). This is coordinate with the observation of stalled neuronal migration in the *Vgat*^*ECKO*^ telencephalon^12^.

### Extensive changes in gene expression profiles in *Vgat*^*ECKO*^ endothelial cells

**Figure 2:**
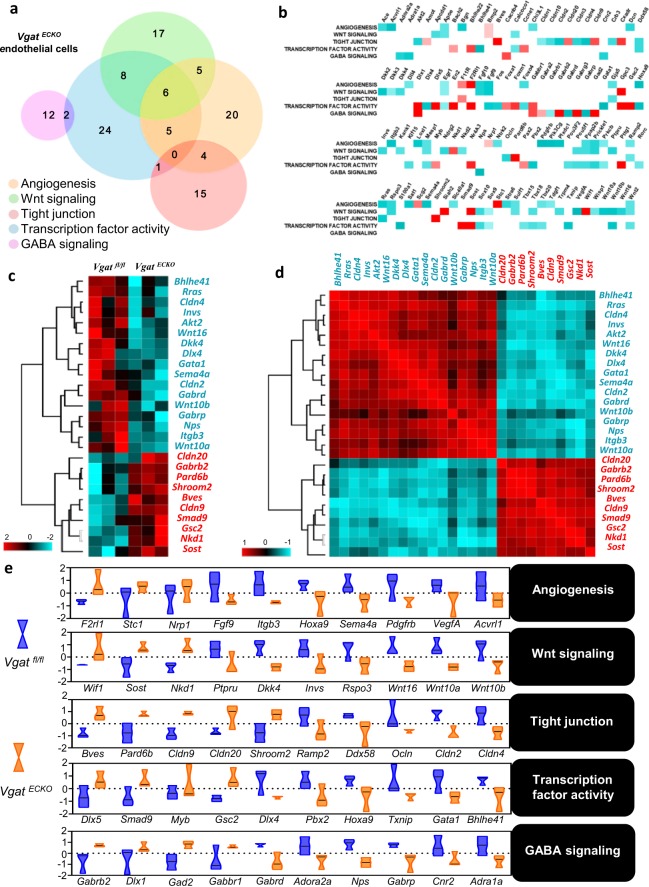
Major gene expression changes in *Vgat*^*ECKO*^ endothelial cells. (**a**) Venn diagram of gene sets overlapping in 5 ‘biological process’ categories of *Vgat*^*ECKO*^ endothelial cells. (**b**) Leading edge analysis of 5 ‘biological process’ gene sets of *Vgat*^*ECKO*^ endothelial cells compared to controls (up-regulation (red color), down-regulation (blue color)). (**c**) Selection and hierarchical clustering of top differentially expressed genes in *Vgat*^*fl/fl*^ and *Vgat*^*ECKO*^ endothelial cells. (**d**) Expression similarity analysis of selected genes by using Pearson correlation method. (**e**) Violin plot comparison of differentially regulated genes (10 genes) in 5 ‘biological process’ categories between *Vgat*^*fl/fl*^ endothelial cells (blue) and *Vgat*^*ECKO*^ endothelial cells (orange).

To obtain detailed biologically relevant information of gene expression dynamics in *Vgat*^*ECKO*^ endothelial cells, we performed step-by-step characterization of the data using multi-faceted approaches. GO enrichment analysis of *Vgat*^*ECKO*^ periventricular endothelial cells revealed 119 differentially expressed genes that were involved in 5 categories related to: angiogenesis, tight junction, Wnt signaling, transcription factor activity and GABA signaling (Fig. 2a). Analysis of the overlap in similarities and differences between gene sets showed that 11 angiogenesis related genes were shared with transcription related genes. Among the 11 genes, 6 genes overlapped with Wnt signaling genes. We next performed a leading-edge analysis with gene sets from the 5 biological categories and found that the enrichment score of most genes from 4 categories including angiogenesis, Wnt signaling, tight junction, and transcription, were significantly low, while some GABA signaling related genes of periventricular endothelial cells showed a higher enrichment score (Fig. 2b). These results implied that angiogenesis and its related biological processes such as Wnt signaling, tight junctions, and transcription, were closely associated, and moreover down-regulated together following the loss of *Vgat* function in endothelial cells. Genes that showed high enrichment score in GABA signaling category (*Gabbr1, Gabra2, Gabrb2, Gabrg3*) may indicate compensational mechanisms in response to the lack or low extracellular GABA signals. To further investigate these gene sets, we performed computational marker selection analysis and identified the top 27 marker genes (Fig. 2c). Genes of Wnt signaling and tight junction categories mostly emerged from the analysis. All selected genes were hierarchically clustered (Fig. 2c) and were displayed in a heatmap matrix for their expression pattern similarity analysis (Fig. 2d). Distinct patterns of gene expression emerged, and high co-relations were found for several gene sets. For instance, expression pattern similarity analysis showed significant upregulation of *Gabrb2, Pard6b*, and *Shroom2*, and significant down-regulation of *Gabrp*, *Nps*, *Itgb3*, and *Wnt10a* in *Vgat*^*ECKO*^ periventricular endothelial cells. Additionally, detailed expression of 10 marker genes in 5 categories: angiogenesis, Wnt signaling, tight junction, transcription factor and GABA signaling in *Vgat*^*fl/fl*^ versus *Vgat*^*ECKO*^ endothelial cells were represented as violin plots (Fig. 2e). An increase in genes necessary for morphogenesis, survival, vascular responses and inflammation (*F2rl1*, *Stc1*, *Nrp1*, *Shroom2*) and a reduction in genes specific for endothelial cell proliferation (*Pdgfrb*, *Vegfa*), differentiation (*Hoxa9*), migration (*Itgb3*, *Sema4a*, *Vegfa*), vascular sprouting and integrity (*Fgf9*, *Vegfa*) was observed in *Vgat*^*ECKO*^ endothelial cells. Gain or loss of function of Wnt pathway signaling components can result in abnormal vascular development and angiogenesis and this was re-capitulated by differential gene expression in *Vgat*^*ECKO*^ endothelial cells. Wnts play multiple roles in governing cell fate, proliferation, migration, polarity, and death, during development^17^. Several genes involved in Wnt signaling/Wnt signaling pathway (*Ptpru, Dkk4, Invs, Rspo3, Wnt10a, Wnt10b, Wnt16*) were downregulated and negative regulation of Wnt signaling pathway genes (*Wif1, Sost, Nkd1*) were upregulated in *Vgat*^*ECKO*^ endothelial cells when compared to controls (Fig. 2e). Disruption of tight junctions disturbs endothelial barrier functions. In the mouse brain, a functional blood-brain barrier (BBB) is formed by embryonic day 15 and endothelial cell-cell junctions provide stable connections to prevent leakage^18^. Differential expression of several claudins, occludin and tight junction associated signaling molecules was observed in *Vgat*^*ECKO*^ endothelial cells (Fig. 2e). Claudin genes (*Cldn2, Cldn4, Cldn9, Cldn20*) related to tight junction formation were significantly altered in *Vgat*^*ECKO*^ periventricular endothelial cells. And *Akt2*, important for maintenance of BBB integrity was downregulated^19^, while *shroom2* that interacts with ZO-1 at tight junctions was up-regulated^20^. Transcriptional regulatory networks were also significantly altered in *Vgat*^*ECKO*^ endothelial cells that can contribute to abnormal blood vessel development. For instance, *Dlx5, Smad9, Myb* and *Gsc* were upregulated, while *Pbx2, Hoxa9, Gata1* and *Bhlhe41* were downregulated. *Hoxa9* transcriptionally regulates the *EphB4* receptor to modulate endothelial cell migration and tube formation^21^ while *Gata1* regulates angiogenic factor *Aggf1* and endothelial cell function^22^. In the GABA signaling category, GABA_A_ receptor subunits expression was altered (*Gabrb2*, *Gabbr1*, *Gabrbp*) in *Vgat*^*ECKO*^ endothelial cells. The homeobox gene, *Dlx1* and glutamic acid decarboxylase isoform, *Gad2*, were concurrently upregulated, but not *Gad1*. GABA is synthesized from glutamate by Gad isoforms. Specific promoter regulatory elements of *Gad2* are direct transcriptional targets of *Dlx1*. This indicates that intracellular GABA levels are ramped up in *Vgat*^*ECKO*^ endothelial cells. Taken together, these results implicate *Vgat* as a critical modulator of multiple angiogenesis pathways in the embryonic forebrain.

### Extensive changes in gene expression profiles in neuronal cells from *Vgat*^*ECKO*^ telencephalon

**Figure 3:**
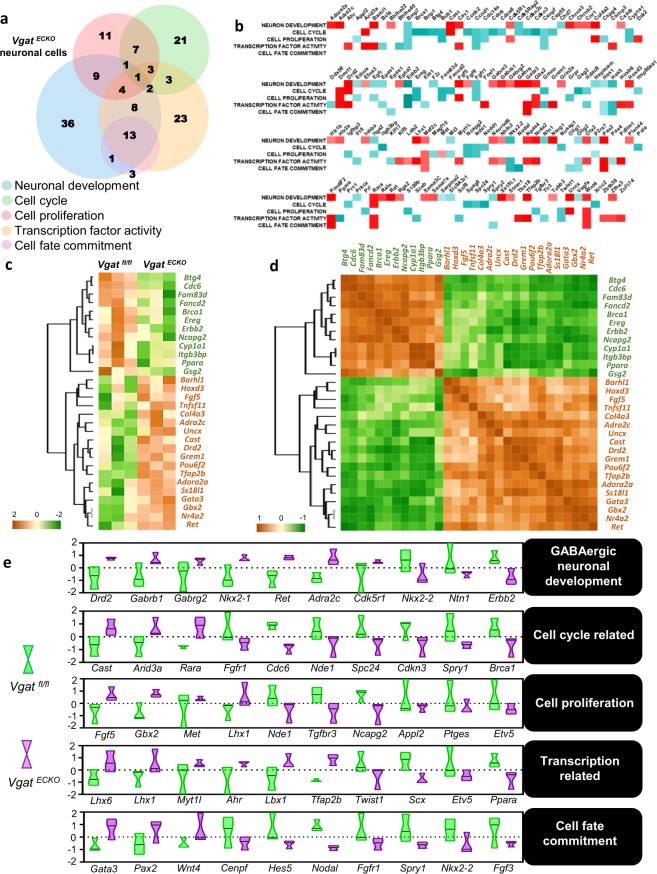
Major gene expression changes in *Vgat*^*ECKO*^ neuronal cells. (**a**) Venn diagram of gene sets overlapping in 5 ‘biological process’ categories of *Vgat*^*ECKO*^ neuronal cells. (**b**) Leading edge analysis of 5 ‘biological process’ gene sets of *Vgat*^*ECKO*^ neuronal cells compared to controls (up-regulation (red color), down-regulation (blue color)). (**c**) Selection and hierarchical clustering of top differentially expressed genes of *Vgat*^*fl/fl*^ and *Vgat*^*ECKO*^ neuronal cells. (**d**) Expression similarity analysis of selected genes by using Pearson correlation method. (**e**) Violin plot comparison of differentially regulated genes (10 genes) in 5 ‘biological process’ categories between *Vgat*^*fl/fl*^ neuronal cells (green) and *Vgat*^*ECKO*^ neuronal cells (purple).

We next turned our attention to understand the gene expression changes in *Vgat*^*ECKO*^ neuronal cells in the absence of endothelial GABA release. Grouping of genes into Venn diagrams illustrated 146 genes in neuronal development, cell cycle, cell proliferation, cell fate commitment and transcription related categories (Fig. 3a). In leading edge analysis, we found that the enrichment score of genes associated with the cell cycle and cell proliferation categories were mostly low, and cell fate commitment were moderately low, while the score of genes related to neuronal development and transcription were moderately high (Fig. 3b). These results denote that altered GABA signaling from *Vgat*^*ECKO*^ periventricular endothelial cells can directly affect several aspects of neuronal development including cell cycle, proliferation, fate commitment and transcription factor activity. We then isolated 30 marker genes for these 5-biological processes in *Vgat*^*ECKO*^ neuronal populations (Fig. 3c). These genes were also clustered hierarchically, and we analyzed expression pattern similarity as a heatmap matrix (Fig. 3d). The analyses revealed prominent changes in genes specifically associated with GABAergic neuronal development, cell cycle, proliferation, fate commitment, and transcription related categories. Genes that displayed co-relation in expression pattern within the upregulated genes set were *Adora2a, Ss18l1, Gata3, Gbx2, Nr4a2* and *Ret*, while in the downregulated genes sets were *Cyp1a1, Itgb3bp* and *Ppara*. Based on the GSEA results, a violin plot was used to represent the differential expression of top 10 genes in the above categories (Fig. 3e). Surface expression of several GABA_A_ receptor subunits (*Gabrb1*, *Gabrg2* {Fig. 3b,e}; *Gabra3, Gabrq* {Fig. 3b}), distinct from the ones on *Vgat*^*ECKO*^ endothelial cells and several genes associated with GABAergic neuronal specification, differentiation and survival (*Nkx2.1*, *Lhx1*, *Lhx6*) were upregulated in *Vgat*^*ECKO*^ neurons. No changes were observed in Gad isoforms (*Gad1*, *Gad2*) or GABA release and uptake mechanisms in neuronal cells (*Gat1*-*4* and *Vgat*). Cell cycle and cell proliferation related genes showed differential expression. While cell cycle genes (*Cdc6, Nde1, Brca1, Spry1, Cdkn3, Spc24, Fgfr1*) were downregulated, positive regulation of cell cycle genes (*Cast, Rara, Arid3a*) were upregulated in *Vgat*^*ECKO*^ neuronal cells. Similarly, while some genes related to cell proliferation (*Etv5*, *Ptges*, *Appl2*, *Ncapg2*, *Tgfbr3* and *Nde1*) were down regulated; some others were upregulated (*Fgf5*, *Gbx2*, *Met* and *Lhx1*). Genes involved in neuronal fate commitment (*Nkx2.2, Hes5, Nodal, Fgfr1, Spry1*) and synapse organization (*Erbb2*), were downregulated in *Vgat*^*ECKO*^ neuronal cells. Neuronal migration associated genes were significantly affected in *Vgat*^*ECKO*^ neuronal populations. Positive regulators of cell motility/migration related genes were significantly downregulated (*Fgfr1*, *Nde1*, *Ntn1* and *Twist1*), while negative regulators of cell migration (*Drd2*, *Adra2c*) were upregulated. For instance, an upregulation of *Drd2* that can slow down neuronal migration was observed. *Drd2* activation has been reported to decrease tangential GABAergic neuronal migration from the ganglionic eminences to the cerebral cortex^23^. These results provide more in-depth mechanistic understanding of the cell proliferation changes and stalled long distance GABAergic neuronal migration in *Vgat*^*ECKO*^ telencephalon^12^. Thus, based on the extensive changes in gene expression patterns, GABAergic neuronal cell gene expression is significantly affected by alteration of endothelial GABA release.

### Validation of gene expression by quantitative real-time PCR analysis

**Figure 4:**
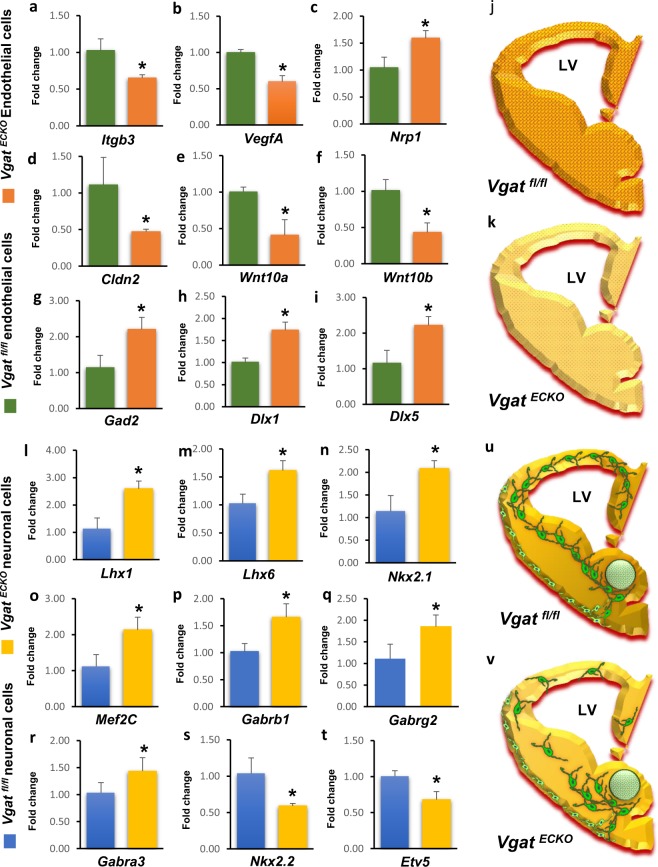
Validation of altered gene expression in *Vgat*^*ECKO*^ endothelial and neuronal cells. (**a**–**i**) RT-qPCR validation of microarray gene expression profiles in *Vgat*^*fl/fl*^ and *Vgat*^*ECKO*^ endothelial cells. Data represents mean ± S.D, (n = 3, *P < 0.05, Student’s t test). (**j**,**k**) Schema depicting altered vascular profiles in *Vgat*^*ECKO*^ telencephalon. *Vgat*^*fl/fl*^ embryonic telencephalon has normal periventricular vascular network (red lattice pattern) and normal endothelial GABA signaling pathway (reddish orange hue) (**j**) while in *Vgat*^*ECKO*^ telencephalon there is complete loss of endothelial GABA secretion (light yellowish hue) that affects periventricular angiogenesis (dotted red pattern) and vascular pattern formation (**k**). (**l**–**t**) RT-qPCR validation of microarray gene expression profiles in *Vgat*^*fl/fl*^ and *Vgat*^*ECKO*^ neuronal cells. Data represents mean ± S.D, (n = 3, *P < 0.05, Student’s t test). (**u**,**v**) Schema depicting GABAergic neuronal tangential migration (green) in *Vgat*^*fl/fl*^ and *Vgat*^*ECKO*^ telencephalon. Complete loss of endothelial GABA secretion (light yellowish hue) has significant consequences for GABAergic neuronal migration resulting in neuronal reductions and abnormal cortical distribution in *Vgat*^*ECKO*^ telencephalon (**v**). LV: lateral ventricle.

We further confirmed some of the notable changes in gene expression in *Vgat*^*fl/fl*^ and *Vgat*^*ECKO*^ endothelial cells and neuronal cells by performing quantitative real-time polymerase chain reaction (qRT-PCR). In *Vgat*^*ECKO*^ endothelial cells, angiogenesis genes, *Itgb3*, and *Vegfa* were down-regulated while *Nrp1* was upregulated (Fig. 4a–c). Vegfa binding to Nrp1 is a trigger for several of the biological functions of *Vegfa*^24^; therefore, the inverse co-relation in *Vegfa*-*Nrp1* expression in *Vgat*^*ECKO*^ endothelial cells is particularly interesting. Tight junction related gene (*Cldn2*) and Wnt signaling genes (*Wnt10a*, *Wnt10b*) were significantly downregulated (Fig. 4d–f). The signaling pathways triggered by *Wnt10a* and *Wnt10b* in embryonic brain endothelial cells are yet uncharacterized. *Cldn2* has been reported as an essential component of tight junctions at the blood retinal barrier and blood-CSF barrier and is believed to contribute to key functions at this interface^25^, and may have new roles in periventricular angiogenesis at this embryonic stage. Another novel aspect is the change in GABA related gene expression in *Vgat*^*ECKO*^ endothelial cells. The absence of a GABA release mechanism triggered an elevated expression of transcription factors *Dlx1* and *Dlx5*, and GABA synthesizing enzyme *Gad2* in *Vgat*^*ECKO*^ endothelial cells (Fig. 4g–i). *Gad* genes are direct *Dlx* transcriptional targets that may explain the concurrent up-regulations. Thus, validation of gene expression changes in *Vgat*^*ECKO*^ endothelial cells provided new mechanistic insights that account for the vascular deficits observed in the *Vgat*^*ECKO*^ telencephalon (Fig. 4j,k).

In the *Vgat*^*ECKO*^ neuronal cell population, we found an increase in the mRNA level of *Lhx1, Lhx6, Nkx2.1* and *Mef2c*, multi-functional determinants of GABAergic neuronal development (Fig. 4l–o), including differentiation, migration, interneuron fate specification and survival. For instance, *Lhx6* and *Nkx2.1* were coordinately upregulated in *Vgat*^*ECKO*^ neuronal cells. *Lhx6* functions directly downstream of *Nkx2.1* in the specification of parvalbumin and somatostatin interneuron fate^26^. *Nkx2.1* expression needs to be normally turned off during interneuron migration to the cortex and increases in *Nkx2.1* expression can lead to an accumulation of MGE-derived interneurons in the striatum^27^ as observed in the *Vgat*^*ECKO*^ telencephalon^12^. The increase in surface expression of several neuronal GABA_A_ receptors units in the absence of endothelial GABA release was also confirmed (Fig. 4p–r). Transcription factor *Nkx2.2* expression that partially overlaps with the expression domains of *Nkx2.1* and *Dlx*^28^ was down-regulated (Fig. 4s), so too was *Etv5* that is important for dendrite development and plasticity^29^ (Fig. 4t). These results provide new mechanistic understanding of the stalled GABAergic neuronal migration and accumulation in the *Vgat*^*ECKO*^ ventral telencephalon (Fig. 4u,v) and abnormal distribution and cortical layer-specific reductions in interneuron populations^12^. Taken together, our data illustrate validated down-stream gene expression changes in endothelial and neuronal cells due to the loss of autocrine and paracrine GABA signaling from endothelial cells.

### Cell type specific gene expression and its significance for neuropsychiatric disease

**Figure 5:**
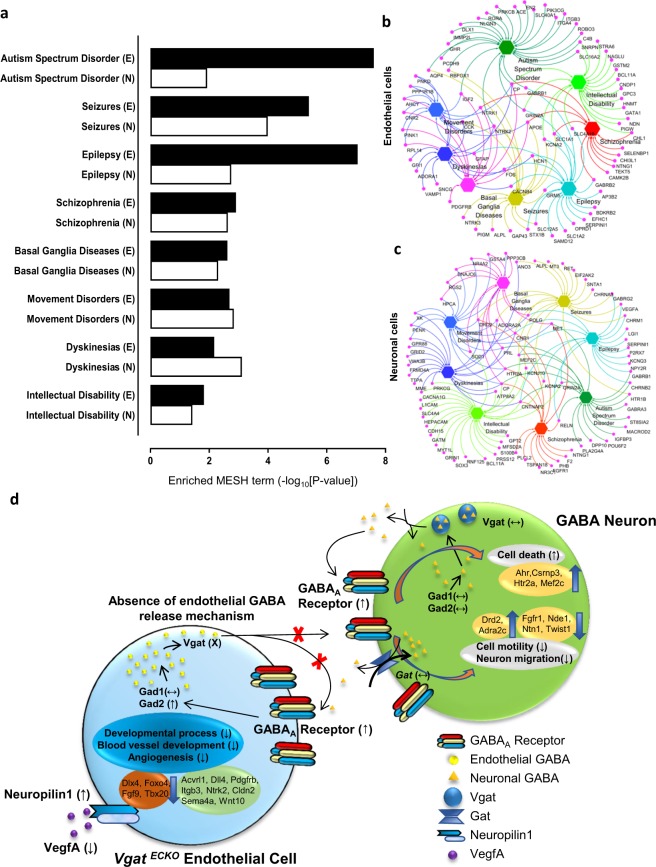
The significance of cell-type specific gene expression for neuropsychiatric disorders. (**a**) Comparison of disease enrichment score between *Vgat*^*ECKO*^ endothelial cells (E) and neurons (N) in similar brain disease categories. (**b**) Network of differentially expressed genes and corresponding brain disorders of *Vgat*^*ECKO*^ endothelial cells based on CTD analysis. (**c**) Network of differentially expressed genes and corresponding brain disorders of *Vgat*^*ECKO*^ GABAergic neurons based on CTD analysis. (**d**) Summary schema highlighting the extensive gene expression changes in endothelial cells and neuronal cells in the absence of *Vgat* and an endothelial GABA release mechanism at embryonic stages.

We next questioned whether the gene expression profile in endothelial cells versus neuronal cells could be used to isolate which cell type contributes to the postnatal phenotype of the *Vgat*^*ECKO*^ mice. Interestingly, when genes were classified according to disease categories by using the CDT database, endothelial cells showed more significant enrichment in autism spectrum disorder and epilepsy categories versus neuronal cells (Fig. 5a), that co-related with the *Vgat*^*ECKO*^ postnatal phenotype. *Vgat*^*ECKO*^ mice show seizure-like activity from P7 onward, with periods of quiescence, tremors and decreased voluntary movement and serves as model for childhood epilepsy or autism^12^. Neuronal cells on the other hand showed similar or greater enrichment in disease categories: intellectual disability, basal ganglia diseases, dyskinesia, movement disorders and schizophrenia (Fig. 5a). We also performed a network analysis to reveal the relationship between diseases and their corresponding genes in *Vgat*^*ECKO*^ endothelial cells and neurons. In gene-disease network relationships, we observed that the genes were distinct with respect to cell types and disease categories (Fig. 5b,c). For instance, autism spectrum disorder related genes were different in endothelial cells versus neuronal cells. Several genes from endothelial cells (*Cp, Apoe, Grin2A, Slc1a1, Ntrk1, Ntrk2, Igf2, Hcn1, and Kcna2)* and neuronal cells (*Drd2, Adora2a, Cnr1, Prl, Sod1, Cp, Cntnap2, Kcna2, Grin2a, Met, Polg, Mef2c, Reln and Htr2a)* however showed cross-connectivity and relationship with 3 or more disease categories. Taken together, our results signify the importance of making correct identification of cell-type specific contributions to a specific disease phenotype. Delineation of cell-type specific effects, neuronal versus endothelial, during the developmental window, is important before treatment strategies can be designed in a disease model.

## Discussion

This study provides new insights into the mechanistic contributions of endothelial *Vgat* to forebrain development. Although neuronal GABA signaling has been extensively studied in the embryonic telencephalon, the molecular mechanisms regulating vascular GABA signaling are unknown. Deletion of *Vgat* from endothelial cells disturbed the normal dynamics of endothelial specific gene expression and strongly affected Wnt signaling and tight junction formation markers, supporting novel functions for Vgat in vascular integrity and early blood-brain barrier development. Among the novel genes that were significantly down-regulated by *Vgat* in endothelial cells were *Wnt10a*, *Wnt10b*, *Wnt16*, *Cld2*, *Cld4* and *Ocln*. Additionally, genes involved in endothelial cell proliferation, migration, sprouting and vascular pattern formation (*Pdgfrb*, *Vegfa*, *Hoxa9*, *Itgb3*, *Sema4a* and *Fgf9*) were significantly affected. Our data also illustrates many new components of GABA signaling in endothelial cells (*Dlx1*, *Gad2*, several GABA_A_ receptor subunits, *Nps*, *Cnr2*) that are affected in the absence of *Vgat*. *Vgat* is thus a crucial mediator for maintenance of telencephalic angiogenesis. GABA release from endothelial cells is critical for activating endothelial GABA_A_ receptors and maintaining a positive feedback cycle. When endothelial GABA vesicular storage and release is absent, extracellular GABA levels are concurrently affected. Absence of this endothelial GABA release mechanism increased surface expression of GABA_A_ receptor subunits in endothelial cells and triggered internal changes in angiogenesis pathway genes (Fig. 5d). Absence of endothelial GABA release also affected extracellular GABA_A_ receptor subunit expression on neuronal cells and directly influenced cell cycle and transcription related genes that determine the intrinsic properties of neuronal differentiation, migration, fate and survival (Fig. 5d). Thus, endothelial GABA release and signaling has profound effects in shaping neuronal development.

The spatiotemporal patterns of neuronal GABA_A_ receptor expression are believed to be important in the orchestration of the normal GABA-related regulation of proliferation and migration of neural progenitors in the embryonic brain^30^. There are several lines of evidence of changes in expression or modification of specific subunits and the altered function of certain GABA_A_ receptors during pregnancy and post-partum period that result in altered synaptic transmission^31^. This is particularly interesting, since deletion of endothelial *Vgat*, altered surface expression of several GABA_A_ receptor subunits on both endothelial and neuronal cells, that can also account for the enhanced neuronal network excitability and changes in plasticity observed in the postnatal period^12^. For instance, *Vgat*^*ECKO*^ neuronal cells showed an elevated GABA_A_ receptor beta 1 subunit expression, while very low expression of the mRNA transcript for the GABA_A_ receptor beta 1 subunit has been reported in the normal embryonic forebrain with expected peaks only at postnatal stages P6–P12^1^. Specific sub-unit composition of GABA_A_ receptors are critical for neuronal migration and may constitute a homeostatic mechanism to maintain a steady balance in establishing GABA’s role as excitatory in the embryonic brain versus inhibitory in the postnatal brain.

Our results also implicate genes enriched in *Vgat*^*ECKO*^ periventricular endothelial cells as being specifically contributory to the disease phenotype of the *Vgat*^*ECKO*^ mouse model versus neuronal cells. Alterations of cell-type specific gene expression provide a template for putative disease subtypes that can be associated with clinical symptoms and phenotypes. These findings implicate the importance of understanding cell-type specific contributions in neuropsychiatric disease origin and calls for a change in perspectives that primarily implicates neuronal dysfunctions to these disorders. Thus, endothelial GABA signaling modulates core aspects of vascular and neuronal development, forming a foundation for more complex neuro-vascular interactions. Consumption of GABA-acting drugs may have profound effects on blood vessels and the blood-brain barrier. In this context, it would be interesting to examine the specific effects of vascular GABA-GABA_A_ receptor signaling in the postnatal and adult brain in future studies.

## Materials and Methods

### Animals

Timed pregnant CD1 mice were purchased from Charles River laboratories, MA. Colonies of GAD65-GFP and Tie2-GFP mice were maintained in our institutional animal facility. *Tie2-cre* mice and *Vgat floxed* (*Vgat*^*fl/fl*^) mice were obtained from Jackson Labs. The *Tie2-cre* transgene is known for uniform expression of cre-recombinase in endothelial cells during embryogenesis and adulthood^12,32–34^. To selectively delete *Vgat* in endothelial cells, *Tie2-cre* transgenic mice (males) were crossed to *Vgat*^*fl/fl*^ mice (females) to generate *Tie2-cre; Vgat*^*fl*/+^ mice (males). These were further crossed with *Vgat*^*fl/fl*^ mice (females) to obtain the *Vgat* conditional knock-outs (*Tie2-cre; Vgat*^*fl/fl*^ mice). The day of plug discovery was designated embryonic day 0 (E0). Animal experiments were in full compliance with the NIH Guide for Care and Use of Laboratory Animals and were approved by the McLean Institutional Animal Care Committee.

### Isolation and primary culture of endothelial cells

Embryonic brains were dissected under a stereo-microscope and the telencephalon was removed. Pial membranes were peeled out and discarded. The remaining telencephalon without pial membranes was pooled (periventricular endothelial cells). Purity of endothelial cell cultures was established with endothelial cell markers and determined to be one hundred percent^13,14,35^. Isolation and culture of endothelial cells was performed according to published methodology^14^.

### Isolation and primary culture of neuronal cells

Primary culture of E15 embryonic neurons was performed using modifications of established methods^15,16^. Briefly, embryonic brains were extracted under a stereo microscope and placed in cold PBS. After removal of pial membrane, telencephalon was dissected from each embryonic brain. Telencephalon was minced into 1–2 mm slice in cold PBS. Minced telencephalon was treated with 0.1x trypsin/EDTA at 37 °C for 5 min. Trypsin treatment was stopped by adding FBS-DMEM media followed by DNase I/ FBS-DMEM. Dissociated cells were filtered with a 40 um cell strainer and finally filtered cells were cultured in poly-D-lysine and laminin coated cover slips (Corning) in 24 well culture dishes (50,000 cells per well) in Neurobasal media (Life technologies) with 1x B-27 (Life technologies) and 1x Glutamax (Life technologies) in the presence of BDNF (100 ng/ml) and GDNF (50 ng/ml) for 4 days at 37 °C at 5% CO_2_.

### Gene expression profile analysis

RNA samples were prepared from *Vgat*^*fl/fl*^ and *Vgat*^*ECKO*^ endothelial and neuronal cell cultures from three different brain pools. Total RNA from each cell type was extracted by using the PicoPure RNA Isolation kit (Arcturus) followed by supplier’s protocol. Mouse Gene 2.0 ST Array (Affymetrix) and data normalization for producing gene level expression values, were performed at the Boston University Microarray & Sequencing Resource, Boston, MA by using the implementation of the Robust Multiarray Average (RMA)^36^ in the affy package (version 1.36.1)^37^ included in the Bioconductor software suite (version 2.11)^38^. Normalization of microarray were performed together using the Robust Multiarray Average (RMA) algorithm and a CDF (Chip Definition File) that maps the probes on the array to unique Entrez Gene identifiers. The result is a matrix in which each row corresponds to an Entrez Gene ID and each column corresponds to a sample. The expression values are log_2_-transformed by default. PCA was analyzed and visualized using Transcriptome Analysis Console 4.0 (Affymetrix). Heatmap visualization, hierarchical clustering, and similarity heatmap analysis were performed using Morpheus (Broad Institute, Boston, MA, USA; https://software.broadinstitute.org/GENE-E/), and ranked by t-test statistics (p < 0.05). Violin plot visualization was generated with Z-score using GraphPad Prism v8.0 (GraphPad Software, La Jolla California USA). Gene ontology was performed by using the Database for Annotation, Visualization and Integrated Discovery (DAVID; https://david.ncifcrf.gov/) v6.8 with modified Fisher’s exact test (p < 0.05)^39^ and was visualized using GraphPad Prism software. GSEA and leading-edge analysis was performed using GSEA3.0 (Broad Institute, Boston, MA, USA; http://software.broadinstitute.org/gsea)^40^. Altanalyzer was used for collection of marker genes from expression profiles of *Vgat*^*ECKO*^ endothelial cells and neurons^41^. Comparative Toxicogenomics Database (CTD; http://ctdbase.org/tools/) analysis (p < 0.01) was performed to categorize genes into corresponding diseases^42^. Cytoscape software (v. 3.7.1) was used to network the gene-disease relationship^43^.

### Quantitative real-time PCR

RT was performed by using iScript Reverse Transcription Supermix kit (Bio-Rad). PCR reactions were run on a CFX96 Touch Real Time PCR (Bio-Rad) with SsoAdvanced™ Universal SYBR® Green Supermix (Bio-Rad). Primers for qPCR (*Cldn2*, *Dlx1*, *Dlx5*, *Etv5*, *Gabrb1*, *Gabrg2*, *Gabra3*, *Gad2*, *Gapdh*, *Itgb3*, *Lhx1*, *Lhx6*, *Mef2c*, *Nkx2.1*, *Nkx2.2*, *Nrp1*, *VegfA*, *Wnt10a*, *Wnt10b*) were obtained from Thermo Fisher Scientific. The housekeeping gene *Gapdh* was used as a reference. The relative gene expression among different samples and subsequent fold increase in *Vgat*^*fl/fl*^ versus *Vgat*^*ECKO*^ endothelial cells or GABAergic neurons was determined according to published methodology^44^.

### Statistical analysis

Statistical significance of differences between groups was analyzed by two-tailed Student’s *t* test (Prism; GraphPad software) and has been noted in individual figure legends. Significance was reported at *p* < 0.05.

## Supplementary information

Supplementary Information

## Data Availability

The authors will make the *Vgat*^*ECKO*^ mouse model, data and associated protocols available upon request.
