# Supplementary material for: Mechanistic insights into autocrine and paracrine roles of endothelial GABA signaling in the embryonic forebrain
Source: Sci Rep. 2019 Nov 7;9:16256. doi: 10.1038/s41598-019-52729-x (PMC6838150; doi:10.1038/s41598-019-52729-x)
Supplement: Supplementary file 1 — Supplementary Information [file 41598_2019_52729_MOESM1_ESM.pdf]

Supplementary Information

Mechanistic insights into autocrine and paracrine roles of endothelial GABA signaling in the embryonic forebrain

Yong Kee Choi[1, 2] and Anju Vasudevan[1, 2]

[1]Department of Psychiatry, Harvard Medical School, Boston, MA-02215
[2]Angiogenesis and Brain Development Laboratory, Division of Basic Neuroscience, McLean Hospital, 115 Mill Street, Belmont, MA-02478, USA.

ϕ Correspondence should be addressed to YK.C (ychoi@mclean.harvard.edu) or A.V. (avasudevan@mclean.Harvard.edu)

Supplementary Figure 1:

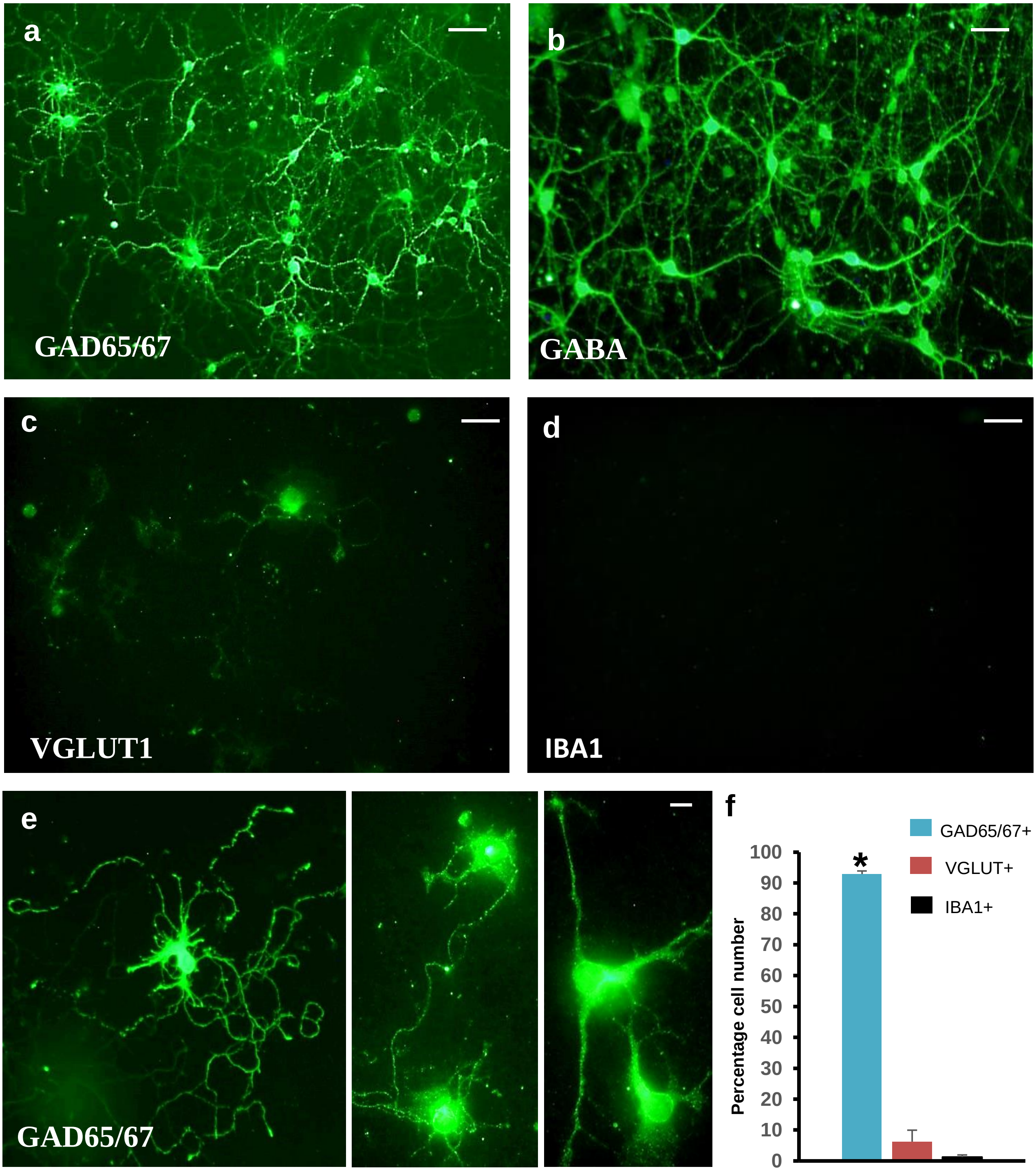

Supplementary Figure 1: (a-f) Immunocytochemical characterization of neuronal populations isolated from E15 control embryonic telencephalon after 4 days of culture with GABAergic neuronal markers (GAD65/67 and GABA; a, b); glutamatergic neuronal marker (VGLUT1; c) and microglial marker (IBA1; d). The culture was enriched with GABAergic neuronal populations with low proportion of glutamatergic neurons (a-d). Microglial cells were virtually absent. (e) High magnification images depict the diverse morphologies of the GABAergic neurons labeled with anti-GAD65/67 antibody. (f) Quantification of GAD65/67^{+}, VGLUT^{+} and IBA^{+} cells in the neuronal culture. Data represents mean ± S.D, (n=3, *P < 0.05, Student's t test; from three independent culture experiments carried out in triplicate). Scale bar: a, 100 µm (applies to b-d); e, 50 um

Supplementary Figure 2:

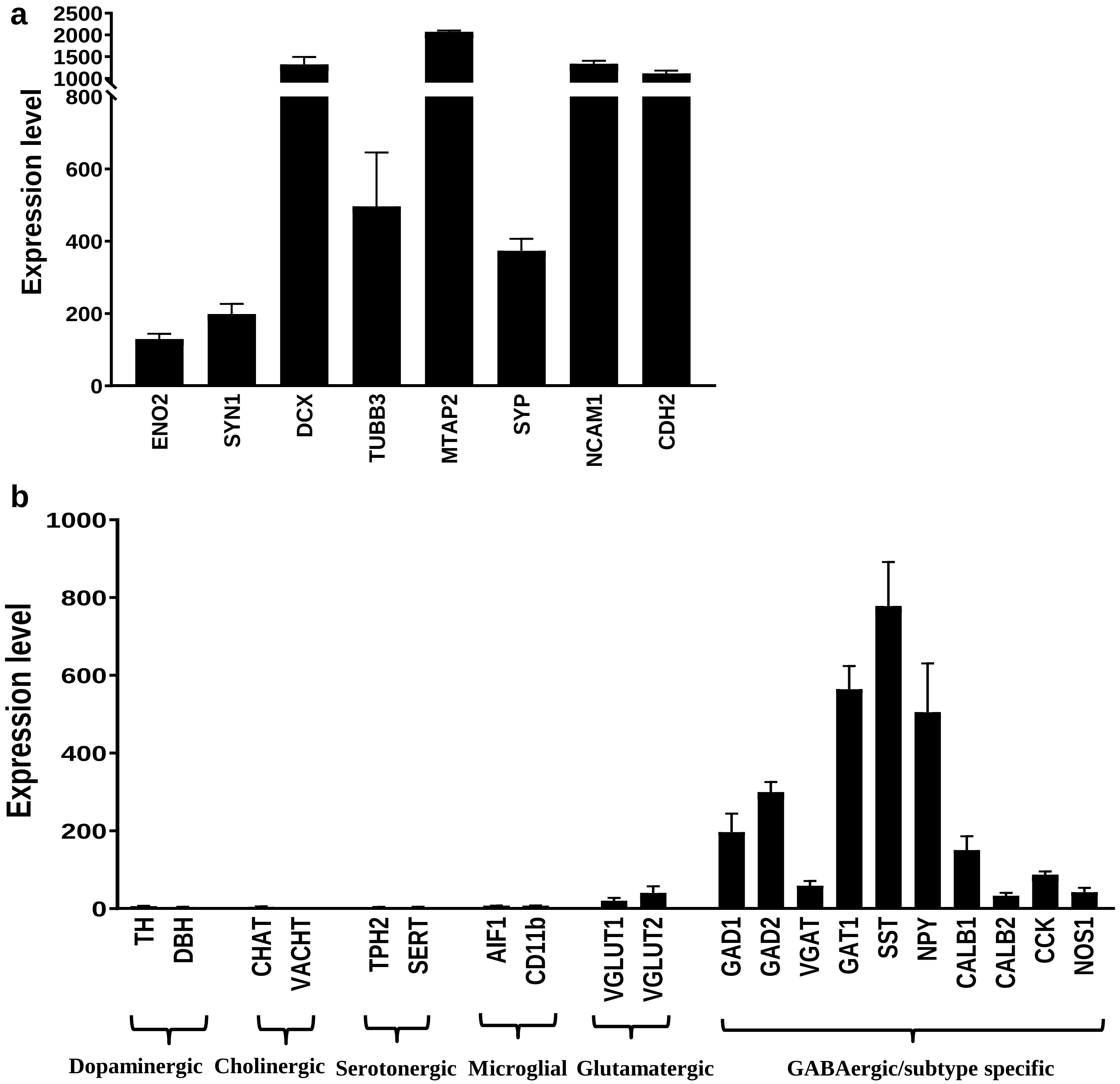

Supplementary Figure 2: (a-b) Gene expression levels of neuronal (a) and neuronal subtype markers (b) in the neuronal population used in this study. An absence of markers for dopaminergic, cholinergic and serotonergic neurons as well as microglial cells was observed. There was low expression of markers for glutamatergic neurons. Significant enrichment of markers for GABAergic neurons/subtypes was observed. Data represents mean ± S.D, (n=3, *P < 0.05, Student's t test).
